# Recent Advances of Chitosan-Based Injectable Hydrogels for Bone and Dental Tissue Regeneration

**DOI:** 10.3389/fbioe.2020.587658

**Published:** 2020-09-17

**Authors:** Guoke Tang, Zhihong Tan, Wusi Zeng, Xing Wang, Changgui Shi, Yi Liu, Hailong He, Rui Chen, Xiaojian Ye

**Affiliations:** ^1^Department of Orthopedic Surgery, Changzheng Hospital, Second Military Medical University, Shanghai, China; ^2^Department of Spine Surgery, The Affiliated Zhuzhou Hospital of Xiangya School of Medicine, Central South University (CSU), Hunan, China; ^3^Department of Orthopedics, Tongren Hospital, Shanghai Jiao Tong University School of Medicine, Shanghai, China; ^4^Beijing National Laboratory for Molecular Sciences, Institute of Chemistry, Chinese Academy of Sciences, Beijing, China; ^5^University of Chinese Academy of Sciences, Beijing, China

**Keywords:** chitosan, injectable hydrogel, responsiveness, bone repair, dental tissue regeneration

## Abstract

Traditional strategies of bone repair include autografts, allografts and surgical reconstructions, but they may bring about potential hazard of donor site morbidity, rejection, risk of disease transmission and repetitive surgery. Bone tissue engineering (BTE) is a multidisciplinary field that offers promising substitutes in biopharmaceutical applications, and chitosan (CS)-based bone reconstructions can be a potential candidate in regenerative tissue fields owing to its low immunogenicity, biodegradability, bioresorbable features, low-cost and economic nature. Formulations of CS-based injectable hydrogels with thermo/pH-response are advantageous in terms of their high-water imbibing capability, minimal invasiveness, porous networks, and ability to mold perfectly into an irregular defect. Additionally, CS combined with other naturally-derived or synthetic polymers and bioactive agents has proven to be an effective alternative to autologous bone and dental grafts. In this review, we will highlight the current progress in the development of preparation methods, physicochemical properties and applications of CS-based injectable hydrogels and their perspectives in bone and dental regeneration. We believe this review is intended as starting point and inspiration for future research effort to develop the next generation of tissue-engineering scaffold materials.

## Introduction

Bone, composed of collagen and calcium phosphate apatite crystals, is a well-known internal support system in higher vertebrates, which provides the rigidity, strength and a certain degree of elasticity to the living body. In recent years, on account of the increase of population aging, accidental injury, disease, trauma, obesity and weak physical activity in internal and external mediators, bone disorders and diseases are on the rise worldwide (Amin et al., 2014; Saravanan et al., 2016; Baldwin et al., 2019; Wang et al., 2019; Yu et al., 2020). Although natural curing is a stable and reliable process, Patients with bone traumas always suffer from impaired healing and rehabilitation. Traditional healing strategy includes the autografts, allografts and xenografts that are employed as bone substitutes to promote bone repair. However, these grafts have many disadvantages in repetitive manipulation process, high cost, surgery wound, immune rejection and potential infectious diseases (Visser et al., 2019). The advent of Bone Tissue Engineering (BTE) brings about the advanced development of bone regeneration at the defect host site without any post-operative complications (e.g., morbidity and immunogenicity). BTE is structured around four key components: (1) osteoblasts generate a bone tissue matrix; (2) biocompatible backbone mimic the extracellular matrix (ECM); (3) vascularization process offer nutrients and wastes transport and (4) morphogenesis signals guide the cell activation (Amini et al., 2012; Guo and Ma, 2014). Thereby, bone tissue engineering material requires favorable properties (e.g., osteoinduction and osseointegration), which can promote the progenitor cell differentiation to osteoblasts, support bone growth and facilitate bone fusion to form new bone tissue. In addition, BTE materials should have chemical and mechanical stabilities, non-thrombosis, easy sterilization and facile manufacturability in the host environment. For example, alveolar bone defects are urgently needed to be regenerated by relying on the advanced BTE materials to bring about a positive impact on dental tissue engineering for the periodontal therapy (Hench and Polak, 2002; Stevens, 2008).

**FIGURE 1:**
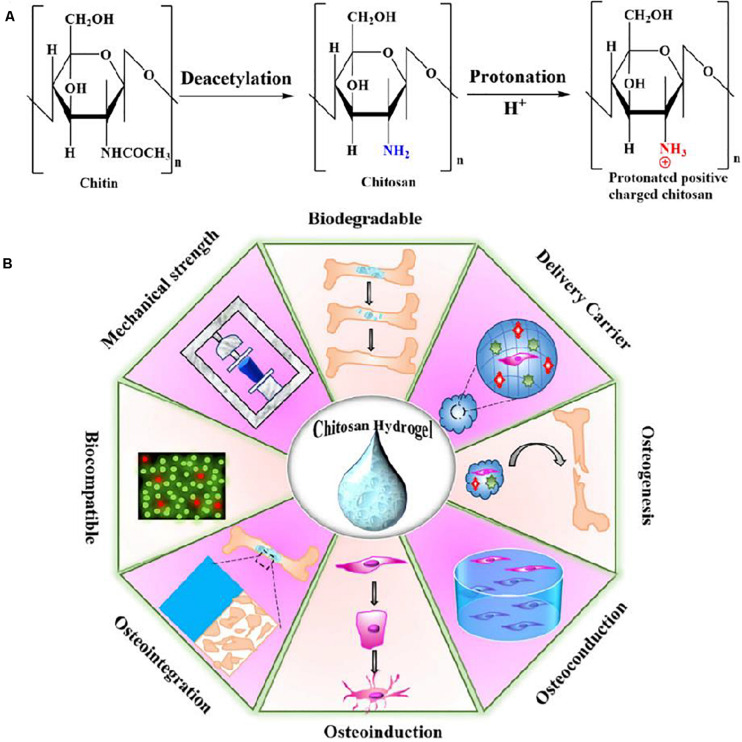
**(A)** The chemical structures for the chitin, noncharged form of chitosan and protonated positive-charged chitosan. **(B)** Schematic representation of physio-chemical and biological properties of CS-based hydrogel. Reproduced from Lavanya et al. (2020) with permission from Copyright 2020 Elsevier.

Natural polymers with good biocompatibility and biodegradability have a variety of characteristics and advantageous properties for living tissues and cells (Li et al., 2020a). As a representative, chitosan (CS), deacetylated form of chitin (Figure 1), is a naturally linear cationic heteropolymer extracted from the shrimp or crab shells. It has analogous compositions and structures to glycosaminoglycans, and renders high biocompatibility, good biodegradability and minimal immune response to tissues and cells (Kavya et al., 2013; Ran et al., 2016; Yang et al., 2016; Atak et al., 2017; Keller et al., 2017). Its physical properties are mainly relied on the molecular weight, degree of deacetylation and purity (Ngo and Kim, 2014). For example, owing to the cationic attribute, CS possesses outstanding antimicrobial activity against both Gram-positive and Gram-negative bacteria, which is relied on the type and degree of deacetylation of chitosan as well as the other extrinsic environmental conditions, but its antimicrobial mechanism has not yet been fully understood. Due to the presence of protonated amino groups of the D-glucosamine residues (Figure 1A), CS can form a non-Newtonian, shear-thinning fluid in most diluted acidic solutions at a pH below 6.5 (pKa value ∼6.3), and further contributes to the complexes with metal ions, polymers, lipids, proteins, DNA, etc. (Berillo et al., 2012, 2014; Zhou et al., 2015; Saravanan et al., 2016; Berillo and Cundy, 2018; Yang et al., 2018; Liu H.Y. et al., 2020). In addition, CS-based hydrogels can be chemically crosslinked by glutaraldehyde, oxidized dextran or other carbohydrates and genipin due to the reductive amination between the amino and aldehyde groups in mild conditions (Berillo et al., 2012; Akilbekova et al., 2018; Lu et al., 2018; Chen et al., 2019). Although CS hydrogel has many advantages, its mechanical properties are weak. Thus, it should be combined with other functional materials to promote the osteogenic differentiation and tissue regeneration (Deepthi et al., 2016; Saravanan et al., 2016; Bao et al., 2020).

## CS-Based Hydrogels

Hydrogel has a three-dimensional porous and interconnected structure that can create a biocompatible ECM microenvironment for cell attachment and proliferation, which can be served as a potential bone supportive system to enhance remodeling at defect sites (Ko et al., 2013; Liu et al., 2017; Wang et al., 2017a, 2020b; Xu et al., 2020a,b). On account of the long and painful invasive surgery for the clinical application of implanted hydrogels, injectable hydrogels are required to have capacities to fill irregularly shaped defects in a non-invasive way, which are recognized as a high-quality and less invasive alternative surgical strategy in BTE (Li et al., 2020b). When the precursor solution containing drugs, cells and/or biomolecules is injected into the subcutaneous region of defective site, composite hydrogels can be quickly formed via the physically or chemically crosslinking reaction with close correlations to the surrounding environments (Yang J.A. et al., 2014; Cao et al., 2018; Wang et al., 2018a; Bao et al., 2019; Liu B.C. et al., 2020). Especially, CS-based injectable hydrogels can be actuated by physical or chemical stimuli via the sol-gel transition. Besides, CS-based injectable hydrogels can deliver cells, drugs and other bioactive molecules, and thus receiving an amount of recognition in fields of cosmetics, food, drug/gene delivery and bone regeneration (Figure 1B; Zheng et al., 2010; Vo et al., 2016; Menon et al., 2018; Yang et al., 2018; Lavanya et al., 2020).

### Preparation Techniques of CS-Based Hydrogels

Hydrogels are generally fabricated via crosslinking the polymer network through physical and chemical strategies. Based on these two basic approaches, there are various preparation techniques for construction of hydrogel configurations. On the one hand, physical crosslinkers mainly include the hydrophobic-hydrophobic, host-guest, ionic or electrostatic, crystallization and stereo complex interactions, which can cause the formation of polymer network in mild conditions (Hoffman, 2012; Sivashanmugam et al., 2015; Fan et al., 2019; Zhu C.X. et al., 2019; Wang et al., 2020a). Physically crosslinked hydrogels possess great advantages in biological applications because of the absence of chemical crosslinkers that may bring about the unpredictable and potential toxicity for the tissues, but their reversible architectures, poor stability and low mechanics greatly limited the scope of applications (Zhang et al., 2013; Maitra and Shukla, 2014; Parhi, 2017; Li et al., 2018). On the other hand, chemical crosslinkers are constructed by the covalent linkage of polymer chains together within the network using high-effective synthetic methods, such as click chemistry, Schiff base reaction, free radical polymerization, etc. (Hennink and van Nostrum, 2012; Wang et al., 2013; Zhang J.F. et al., 2019). On account of the permanent and irreversible junctions among polymeric chains, chemically crosslinking hydrogels possess stable structures and excellent mechanics for tissue engineering fields. However, toxic chemical agents and difficult sterilization may produce adverse effects and certain insecurities.

### Physico-Chemical Properties of CS-Based Injectable Hydrogels

In recent years, the application of injectable in-situ forming hydrogels in orthopedics has been widely investigated by many scientists (Gyawali et al., 2013). Unlike the prefabricated stents that require surgical implantation, injectable hydrogels can be *in situ* injected into the defect sites to fill in any geometric deformities using the minimally invasive surgery methods. Hydrogel is usually used for confronting bone defects in non-weight bearing parts or injured bone tissue through delivering and releasing the therapeutic biomolecules and agents once the stimulus response that triggers its physical and/or chemical properties has been changed. On account of numerous hydroxy and amine groups within the cationic CS framework, CS can be feasibly modified to improve its multifunction, such as antibacterial property, solubility, stimulus-response, adhesion and degradation behaviors (Lavanya et al., 2020). Therefore, CS-based injectable hydrogels possess more advantages on the tailor of topological structures, biodegradability behaviors, cytocompatibility and adhesive force, which can be widespread used as effective biopharmaceutical materials to promote bone regeneration (Liu et al., 2017; Shariatinia and Jalali, 2018).

Chitosan is usually combined with other natural-derived or synthetic biomaterials via the covalent and/or non-covalent bonds, yielding a varied of multifunctional hydrogels. Wherein, physical gelation is a typical approach for fabrication of CS-based hydrogels with good biocompatibility and gradual degradability to promote the cell-materials interactions and stimulate the proliferation and differentiation of osteoprogenitor cells (Berger et al., 2004). Therefore, development of CS-based injectable hydrogels would allow for the effective therapy for the bone regeneration, especially for the irregular defective sites of bone tissue. Based on this physical gelation of CS-based injectable hydrogel, environmentally responsive injectable hydrogels, such as pH, light and temperature, are widely used for repair of large bone defect, because an externally applied trigger for gelation can easily tailor sol-gel transition with the facile permeation into the defect sites and quick gelation *in situ* to fully seal the injury. Recently, Li et al. (2015) prepared a series of soluble UV-crosslinkable CS-based injectable hydrogels by modification of amine groups of CS with methacrylic anhydride in the absence of any activators. These methacryloyl groups improved the CS solubilization in water by impairing intra-/intermolecular hydrogen bonds and provided the crosslinkable CS derivatives. Xia et al. (2017) reported a photopolymerized injectable water-soluble maleilated chitosan (MCS)/poly(ethylene glycol) diacrylate (PEGDA) hydrogels, which had faster gelation rate, higher compressive strength than MCS hydrogel under UV radiation. Control of the MCS/PEGDA ratio could tailor the swelling behavior and mechanical properties of composite hydrogels that could promote the L929 cells attachment and tissue regeneration. Instead of UV light, Hu et al. (2012) prepared CS hydrogels crosslinked under visible light for tissue engineering. By tailoring three various blue light initiators (camphorquinone, fluorescein and riboflavin), methacrylate glycol CS can form the CS hydrogels under the visible light (400–500 nm at 500 mW cm^–2^), displaying good mechanical properties and less damages to cells than energetic UV light. Further, diverse therapeutic agents or drugs can be encapsulated into these temperature and pH-responsive injectable hydrogels to form the multifunctional hydrogels in tissue engineering and drug delivery systems. For example, Qi et al. (2013) incorporated the bone morphogenetic proteins (BMPs) and mesenchymal stem cells (MSCs)/osteoprogenitors into CS-based injectable hydrogels. The thermo-sensitive CS/poly (vinyl alcohol) (PVA) composite hydrogel can offer the strength support and tailored degradation time, which induced the tissue repair via the space conduction and occupation of defect part. In addition, compared to the pure CS hydrogel, introduction of non-degradable PVA could obviously postpone the degradation and prolong the self-healing term of tissue within the defect center.

**FIGURE 2:**
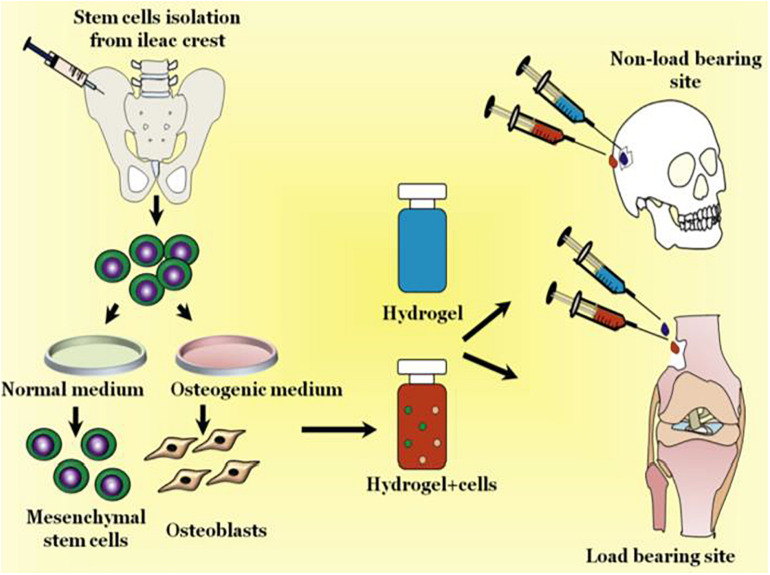
Schematic representation of injectable hydrogels for treating bone loss at defective sites. Reproduced from Saravanan et al. (2019) with permission from Copyright 2019 Elsevier.

Besides, rheological properties also play vital roles in injectable CS-based hydrogels, especially for the gelation, firmness and durability of crosslinking network, which provide intriguing guidance on the effective selection of appropriate chitosan for wide application. Many studies on rheological properties of chitosan in solution have been conducted to characterize the effects various parameters such as polymer concentration, degree of deacetylation, concentrated chitosan, chitosan and vinyl polymers, temperature, shearing and storage time, and addition of other polymers/nanoparticles on the viscoelastic behaviors, which are closely relative to a peculiar mechanism of association with the double-chain strands connected network (Hwang and Shin, 2000; Chen et al., 2003; Martiínez et al., 2004; Lewandowska, 2009; El-hefian and Yahaya, 2010; Racine et al., 2017). Until now, injectable hydrogels are widely applied for tissue engineering and orthopedic applications. Unlike surgical implantation methods, pre-fabricated hydrogel can be injected into any geometries of defect bearing sites using minimally invasive procedures and quick *in situ* gelation through a sol-gel transition once exposure to a simulative change. In addition, injectable hydrogels can also be blended with other biomolecules, therapeutic agents and cells to form multifunctional carriers to repair the bone and dental tissues (Figure 2; Saravanan et al., 2019).

### pH-Responsive CS-Based Injectable Hydrogels

Chitosan and its derivatives are a series of natural biopolymers with good biocompatibility, pH sensitivity, enzymatic biodegradability and polycationic attributes. Especially, tailor the protonation/deprotonation of –NH_2_ group can obviously furnish CS-based injectable hydrogels with pH-responsive behaviors (Xu and Matysiak, 2017). Lower pH can protonate amine group to induce the electrostatic repulsion, and thus the polymer chains can easily expand and interact with water molecules to effectively improve the water-solubility. In contrast, higher pH can deprotonate the -NH_2_ group and cause the collapse of globular structure, severely impairing the water-solubility of CS. In this case, the water solubility and swelling property of CS-based hydrogels is mainly relied on the its pKa value and external pH conditions (Rizwan et al., 2017). Therefore, CS-based injectable hydrogels possess the innate pH response that has been attracted considerable attentions in the bone regeneration process. In addition, incorporation of various polymers with CS can improve this pH responsiveness. Nevertheless, CS has poor solubility in alkaline and neutral solutions as well as the insufficient mechanical performance. To overcome these limitations, various physical or chemical modification strategies have been well-developed to make CS soluble without impairing its other properties.

**FIGURE 3:**
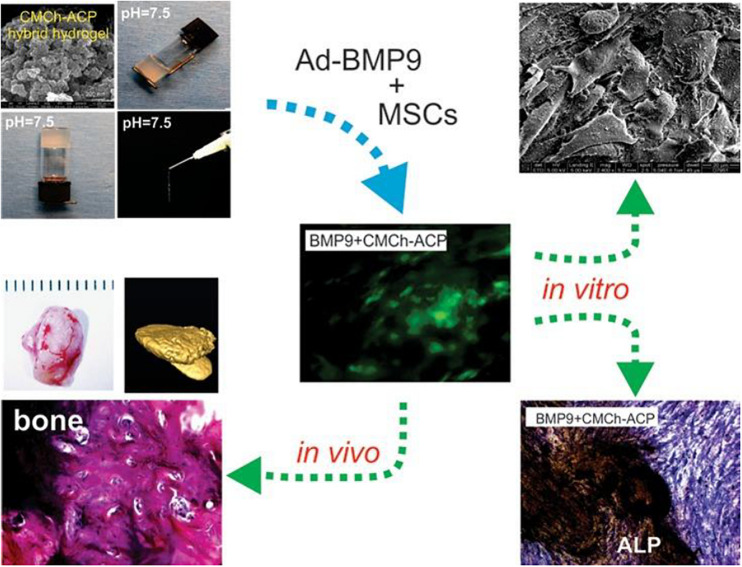
Schematic representation of pH-responsive CS-based hydrogels for bone tissue engineering. Reproduced from Zhao et al. (2019) with permission from Copyright 2019 American Chemical Society.

For instance, Rogina et al. (2017) demonstrated a pH-responsive CS-hydroxyapatite hydrogel with a gelling agent of NaHCO_3_. Tailoring of NaHCO_3_ can achieve the quick gelation of CS-hydroxyapatite-based hydrogel within 4 min, which exhibited good viability for cell proliferation and differentiation as a potential cell carrier. Zhao et al. (2019) developed a composite CMCh-ACP hydrogel via combining the carboxymethyl chitosan (CMCh) and amorphous calcium phosphate (ACP). Incorporation of an acidifier of glucono δ-lactone could endow pH-responsive injectable hydrogels with good biocompatibility and effective cell adhesion/proliferation, which upregulated the expression of bone markers and enhanced the new bone regeneration (Figure 3).

### Thermo-Responsive CS-Based Injectable Hydrogels

**FIGURE 4:**
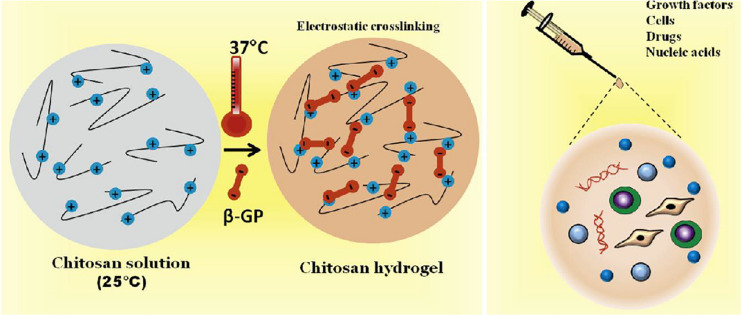
Schematic representation of thermal-responsive CS/β-GP hydrogels encapsulating with various growth factors, cells, drugs and nucleic acids for bone tissue engineering. Reproduced from Saravanan et al. (2019) with permission from Copyright 2019 Elsevier.

Chitosan itself has not thermosensitive behaviors, and thermo-responsive CS-based hydrogels are generally fabricated via introducing the thermo-responsive polymers into CS-based hydrogels (Argüelles-monal et al., 2018). In views of simple regulation and easy utilization for *in vitro* and *in vivo* testing, temperature is recognized as a typical stimulus for the hydrogel system. In recent years, ideally thermo-responsive CS injectable hydrogels with the sol-gel transition at a physiological temperature have been developed in application of tissue engineering because of their temperature response, attractive moldable ability, tailorable rheological property, excellent biocompatibility and biodegradability to the cells and tissues, which can provide suitable manipulation and enhance cellular activity for bone and dental regenerations. In addition, encapsulation of diverse polymers (natural and synthetic polymers), bioactive molecules and nanoparticles can also expand the functional properties of CS injectable hydrogels for biomedical applications (Mohebbi et al., 2019; Zarrintaj et al., 2019; Bagheri et al., 2020; Khalili et al., 2020; Mahmodi et al., 2020; Nourbakhsh et al., 2020). For example, CS/β-glycerophosphate (GP) hydrogel is a famous scaffold for wide-range delivery of growth factors, cells, small drugs and nucleic acid. Since cationic CS chains can be connected with negatively charged GP molecules via the electrostatic attraction and hydrogen bonds, injectable CS/GP hydrogels can achieve structural optimization systems with a sol-gel transition temperature of 37°C for the bone regeneration (Figure 4; Dhivya et al., 2015; Saravanan et al., 2019).

The gelation mechanism of this thermo-responsive CS/GP hydrogel has been detailedly elucidated as follows (Chenite et al., 2001). Firstly, on account of poor solubility of CS in neutral and basic solutions, CS solutions above pH 6.2 can result in the immediate formation of hydrated gel-like precipitate as well as the occurrence of phase separation of CS at pH >6 to form a hydrogel. Maintaining CS solution within a physiological pH (6.8–7.2) in the presence of GP polyol salt could generate a thermo-responsive injectable hydrogel, which kept a sol state at room temperature and transferred into a gel state at physiological temperature (cloud point of ≥37°C). Incorporation of GP components into CS solutions can reduce electrostatic repulsion between phosphate groups of GP and amino groups of CS, enhance CS-CS hydrophobic interactions and increase hydrogen bonding effects between CS chains during gel formation, and therefore the pH range can be modulated from 7.0 to 7.4 and the sol-gel transition temperature can be tailored at 37°C. The temperature dependence of CS/GP hydrogels has been investigated by the rheological studies. Owing to the hydrophobic interactions between CS chains and glycerol groups, CS chains aggregation can be prevented by the CS-water interactions at low temperature. Once upon heating, water molecules are partially removed from the glycerol moieties and CS chains can associate and aggregate into a gel formation. In this CS/GP gelling system, temperature rise can also tailor CS-CS hydrophobic interactions in addition to the regulation of electrostatic forces, because the orientation of dipolar water molecules surrounding CS polymer chains is effectively limited via increasing the vibrational and rotational energy of water molecules. These energized water molecules are removed around the CS polymeric chains, thereby causing the interconnection among dehydrated hydrophobic segments. At low temperatures, CS displays a helical structure due to the presence of intramolecular hydrogen bonds and the masking of physical connections. At high temperature, the decreasing number of intramolecular hydrogen bonds allows the CS molecules to unfold and facilitate the gelation. Thus, pH and temperature are significantly important factors for this thermo-responsive CS/GP injectable hydrogels.

#### Polymers Introducing the CS-Based Injectable Hydrogels

**FIGURE 5:**
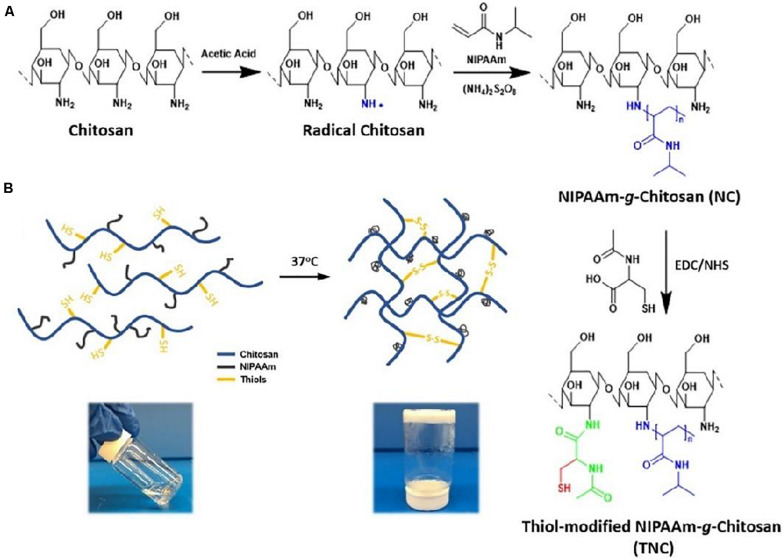
**(A)** Synthetic preparation of NIPAAm-g-CS. **(B)** Gelation mechanism of physical cross-linking (helix-coil structure) and chemical cross-linking (disulfide bonds). Reproduced from Wu et al. (2018) with permission from Copyright 2018 Elsevier.

Incorporation of various polymers like naturally-derived (e.g., alginate, hyaluronic acid, collagen, starch, and silk fibroin) and synthetic macromolecules [e.g., poly(vinylalcohol), polyethylene glycol and polycaprolactone] into CS hydrogels can further improve their multifunctionality and thermo-responsive behaviors for effective bone repair. For example, Bagheri et al. (2019) prepared a kind of electroactive hydrogel based on chitosan–aniline oligomer and agarose, wherein the aniline oligomer can tailor the swelling, degradation rate, thermal and conductive properties of CS-based hydrogels. Owing to its conductivity, control of the electrical stimulus can manipulate the on-demand drug release that may promote the cell activity, growth and proliferation. Chen et al. (2013) prepared a biocompatible injectable thermo-responsive HA-CPN/BCP hydrogel based on a hyaluronic acid-g-chitosan-g-poly (N-isopropylacrylamide) containing biphasic calcium phosphate (BCP) ceramic microparticles. The HA-CPN/BCP possessed better biocompatibility with human fetal osteoblast cell attachment, proliferation and osteoblastic differentiation. In addition, higher mechanical strength and elasticity of these thermo-responsive injected hydrogels was benefited for calcium deposition, extracellular matrix mineralization and generation of ectopic bone tissue. Additionally, incorporation of synthetic polymers endows the CS hydrogels with multifunction to achieve the advanced regulation. Wu et al. (2018) constructed a thermo-sensitive NIPAAm-g-CS hydrogel with disulfide crosslinkers throughout the networks (Figure 5), and found that three types of cells exhibited excellent biocompatibility with favorable cell attachment, growth and proliferation.

#### Bioactive Molecules Encapsulated CS-Based Injectable Hydrogels

Over the last decade, CS-based hydrogels have been functionalized via loading bioactive molecules into drug delivery systems, compelling the bioactive molecules to locally deliver into target sites with adequate dose for a desired period. Encapsulation of hydrophobic and hydrophilic molecules like bioactive factors, drugs, proteins, amino acid, and nucleic acids into CS-based responsive hydrogels can construct smart delivery system and bone regenerative medicine. Wherein, these bioactive molecules [e.g., bone morphogenetic protein 2 (BMP-2) and vascular endothelial growth factor (VEGF)] can keep long stability and half-life with low adverse effects while thermo-responsive injectable hydrogels can effectively prolong the localized drug or growth factor release in a sophisticated delivery system (Tiffany et al., 2012; Faikrua et al., 2014; Mi et al., 2017). In this case, these bioactive molecules-encapsulated CS-based hydrogels can modulate and promote cell differentiation, proliferation, migration, recruitment and angiogenesis. For example, Li et al. (2017) demonstrated a thermo-responsive CS/CSn (pDNA-BMP2)-GP hydrogel system of CS-based hydrogel with pDNA-BMP2 for alveolar bone regeneration. The sustained release of pDNA-BMP2 could affect the osteocytes growth within the lacunae, increase trabecular thickness and promote trabecular bone formation. Apart from these growth factors, CS-based hydrogels could deliver bone resorption inhibitors of alendronate (ALN) to treat the Paget’s disease and osteoporosis. Nafee et al. (2017) prepared ALN-loaded CS/β-GP hydrogel with injectable and thermo-reversible gelation behaviors. These biodegradable and biocompatible hydrogels expressed less inflammatory responses and faster proliferation of granulation tissue via controlling ALN drug release for 65 days.

#### Nanoparticles Encapsulated CS-Based Injectable Hydrogels

Incorporation of functional nanoparticles (NPs) into hydrogels can increase the mechanical, biological, and chemical properties and therefore expand their applications. Especially, metal/metal-oxide NPs (e.g., Au, Ag, and Fe_3_O_4_), inorganic/ceramic NPs (e.g., hydroxyapatite, calcium phosphate, silica and silicates), and polymeric NPs (natural/synthetic polymers, dendrimers and hyperbranched polyesters) can promote the osteogenic differentiation and mineralization for bone regeneration (Moorthi et al., 2014; Wang et al., 2014, 2016, 2017b, 2018b; Yang Y.Y. et al., 2014; Yang et al., 2015, 2021; Thoniyot et al., 2015; Ko et al., 2018; Min et al., 2018; Bian et al., 2020; Fan et al., 2020). For example, Radwan et al. (2020) prepared CS-based calcium phosphate composite scaffolds with embedded moxifloxacin hydrochloride. Composite hydrogels delivered the antibiotic drugs for 3 days and induced the cell differentiation and osteoblasts proliferation, benefiting for bone tissue formations using the osteomyelitis-induced animal model. Moreira et al. (2018, 2019) constructed a novel thermo-responsive CS/gelatin/nBG-based composite hydrogel by combining CS-gelatin with bioactive glass NP (nBG), on account of its capacity to bond with living tissues, these thermo-responsive composite hydrogels possessed enhanced osteoblasts proliferation and angiogenesis-related gene expressions, thus promoting cell proliferation activity and bone regeneration. Additionally, introducing metallic NPs into injectable hydrogels can facilitate bone cell growth and expand bone regeneration applications. For example, when the Au NPs and κ-carrageenan poly(NIPAM) were mixed into the CS-based injectable hydrogels, Pourjavadi et al. (2019) found these Au NPs-incorporated injectable hydrogels were conductive that could enhance cell attachment and proliferation to accelerate bone tissue growth through MG-63 cell viability assay, presenting a great potential application in tissue engineering.

### CS-Based Hydrogels for Bone Regeneration

**FIGURE 6:**
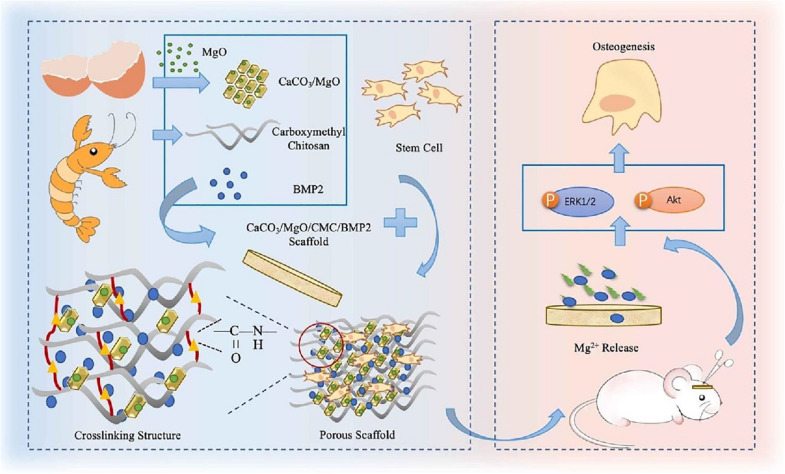
Schematic preparation of CaCO_3_/MgO/CMC/BMP2 scaffolds and their applications *in vivo*. Reproduced from Huang et al. (2020) with permission from Copyright 2020 Elsevier.

Bone tissues are highly functional connective that can constitute the human skeleton, provide the mechanical support, protect internal organs and participate in many physiological activities (Arvidson et al., 2011). Once it is injured, bone tissues can undergo the self-repair process by stimulating MSCs toward osteogenic differentiation and forming the neo-angiogenesis for small size of bone defect, but large-size defects should require bone grafts in the clinical procedures (Gómez-Barrena et al., 2015; Loi et al., 2016). On account of low immunogenicity, physiological inertia, osteoconductivity and osteogenesis, CS-based hydrogels can significantly promote cell proliferation and cell adhesion, presenting great application prospects in bone tissue regeneration (Jiang et al., 2019). In addition, to improve its mechanics and multiple function, CS-based hydrogels are always required to be mixed with other synthetic or natural polymers and bioactive pharmacological molecules to construct multifunctional biomaterials scaffolds. For example, Oudadesse et al. (2020) proposed a nBG-loaded hybrid CS-based hydrogel using freeze-gelation method. The surface area, porosity and mechanical properties of BGN/CH composite hydrogel could be tailored by altering the BGN proportion, which could favor the generation of apatite layer on the surface to promote the direct bone bonding with the implanted hydrogels. Zhang Y.H. et al. (2019) developed a biodegradable hybrid DN hydrogel via interspersing a methacrylated gelatin network into a nanocomposite hydrogel comprised of methacrylated chitosan and polyhedral oligomeric silsesquioxane. Based on its easy manipulation and excellent mechanics, hybrid DN hydrogels could preferentially guide the MSCs toward the osteogenic differentiation *in vitro* and accelerate new bone regeneration *in situ* using a rat of calvarial defects. These advantageous characteristics and attractive capacities endowed these functional DN hydrogels with great potentials for stem cell therapy and tissue engineering application. It is noted that tunable osteogenesis and degradation rates are important for repairing the bone defect. Huang et al. (2020) prepared a novel composite CaCO_3_/MgO/CMC/BMP2 scaffold with high mechanical strength, mineralization ability, biomimetic activity and osteogenic differentiation. The sustainable release of Mg^2+^ and BMP2 played an important role in activating the phosphorylation of ERK1/2 and Akt pathways, which guided the osteogenesis formation using an in-situ rat calvarial defect model (Figure 6).

### CS-Based Hydrogels for Dental Repair

#### Periodontal Regeneration

**FIGURE 7:**
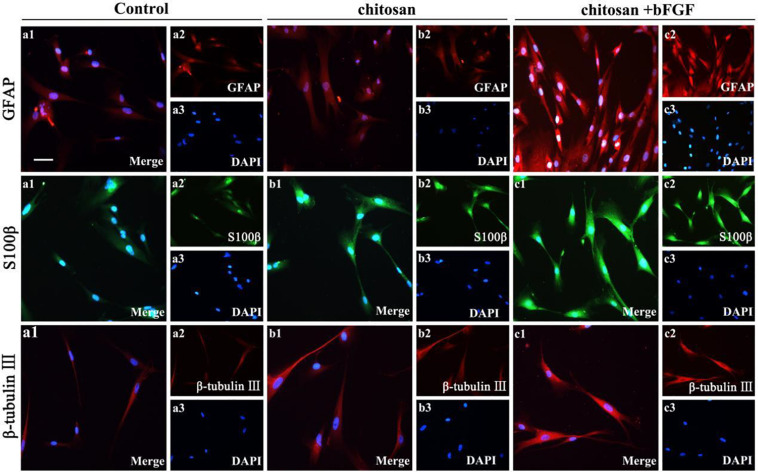
Schematic illustration of the preparation and application of the CS/β-GP/gelatin hydrogels. Reproduced from Xu et al. (2019) with permission from Copyright 2019 Elsevier.

Periodontitis is a chronic inflammation of periodontal support tissues caused by the local bacterial infection. The age of onset was after 35 years old. If gingivitis is not treated in time, the inflammation can spread from gingival to the periodontal membrane and alveolar bone, thus forming the periodontitis. Due to no obvious conscious symptoms and easy to be ignored at initial stage, once the symptoms are more serious, treatment of such pathology is a great challenge for the clinician (Cortellini and Tonetti, 2015; Larsson et al., 2015; Kinane et al., 2017; Roi et al., 2019; Xu et al., 2020a,b). In general, reducing the inflammation is a key to control the infection for periodontal therapy, but traditional mechanical treatments like scaling and root planin are closely connected with the wound repair. In addition, non-surgical treatment alone is insufficient for adjuvant therapy (Abdul Rahman et al., 2019; Harmouche et al., 2019). Therefore, use of local delivery of active drugs has been utilized as an effective strategy to address the inflammation and promote tissue repair (Morand et al., 2017; Batool et al., 2018; Petit et al., 2019). By means of the injectable chitosan hydrogels with modulable physico-chemical properties, CS-based delivery system can be employed as reliable vehicle to release the encapsulated active drugs (e.g., statins, doxycycline, antibiotics, and antiseptics) at the disease site within periodontal pockets (Özdoğan et al., 2018). Simultaneous anti-inflammation and periodontium regeneration is vital for terminating the alveolar bone resorption. Based on this, Xu et al. (2019) proposed a thermo-responsive CS-based injectable hydrogel with the main components of CS, β-sodium glycerophosphate (β-GP) and gelatin. After tailoring the continuous release behavior of aspirin (ASP) and erythropoietin (EPO), CS/β-GP/gelatin@ASP/EPO scaffolds exerted pharmacological roles of anti-inflammation and periodontium regeneration, indicating the suitable candidate for dental treatment in the clinical fields (Figure 7). Zang et al. (2019) also prepared a thermo-sensitive bone morphogenetic protein-7 (BMP-7) and ornidazole (ORN)-encapsulated CS/β-GP composite hydrogel. The BMP-7 and ORN molecules could be stably and sustainably released from the hydrogels, displaying the significantly antimicrobial activity and improved osteoclasts ability than that of control groups. The results indicated that CS/β-GP hydrogels with the controllable delivery of BMP-7 and ORN were valuable and promising for the periodontal therapy.

#### Dental-Pulp Regeneration

**FIGURE 8:**
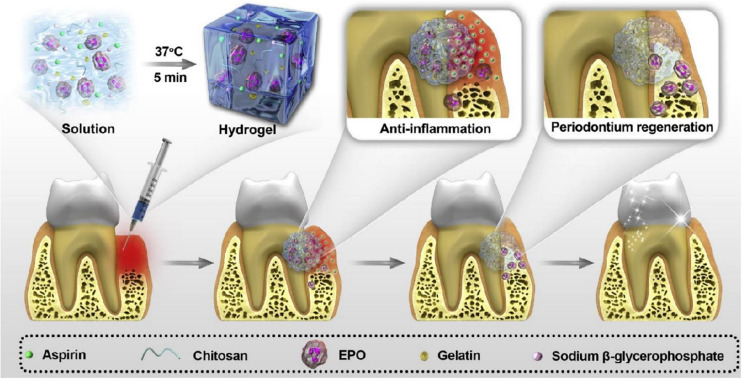
Effect of chitosan/bFGF scaffolds on neural differentiation of DPSCs. Immunofluorescence staining of GFAP, S100β and β-tubulin III. Reproduced from Zheng et al. (2020) with permission from Copyright 2020 Informa Healthcare.

Dental-pulp mainly consists of nerves, blood vessels, lymph and connective tissue, as well as odontoid cells lining periphery of the pulp, aiming to form dentin with the ability of nutrition, feeling and defense. The pulp nerve is particularly sensitive to external stimuli and can produce unbearable pain. In general, dental-pulp regeneration strategy aim to achieve the regeneration of connective tissue, dentin tooth root edification, vascularization and innervation (Keller et al., 2015). Based on the smart drug delivery system, hydrogels are used to deliver active molecules and carry competent cells within the endodontic compartment for the endodontic treatment, which required adequate viscosity and injectable nature of hydrogels to quick adhesion and gelation in the whole root canal system. Recently, it is reported that CS-based hydrogels are widely developed for dental-pulp regeneration, because they can promote proliferation, migration and odontoblastic differentiation of dental pulp stem cells (DPSCs) and MSCs both *in vitro* and *in vivo* (Bordini et al., 2019; Ducret et al., 2019; Zhu N. et al., 2019). For example, Zhang et al. found that when the DPSCs were cultured in CS-based hydrogels containing basic fibroblast growth factor (bFGF) for 7 days, GFAP, S100β and β-tubulin III levels were significantly increased than the control and pure chitosan groups, indicating the non-toxic CS hydrogels carry and transport the therapeutic cells effectively to the recipient area and great potential for the DPSCs survival and dental-pulp regeneration (Figure 8; Zheng et al., 2020).

It is well-known that VEGF plays a crucial role in reparative dentin formation, whereas its constant administrations remains problematic within the body. Wu et al. (2019) conducted a VEGF-loaded injectable CS/β-GP composite hydrogel with a sustained drug release behavior. After cultured DPSCs with CS/β-GP hydrogel, CCK-8 assay found that DPSCs could keep cell adhesion and proliferation activity. Along with the continual release of VEGF from the VEGF/CS/β-GP hydrogel, odontogenic differentiation capacities were significantly improved, which could be utilized as an ideal drug carrier for the pulp capping therapy.

## Future Outlook and Conclusion

Chitosan, a deacetylated form of chitin, is widely utilized as a positive-charged and low-cost natural polymer for bone and dental tissue engineering applications. On account of the excellent biocompatibility, good biodegradability and adjustable physico-chemical properties, in this review, we briefly summarized the various physio-chemical properties of CS-based injectable hydrogels for bone repair and explored in depth the updated progress of CS-based hydrogels with more innate advantages to promote cell attachment, spreading, proliferation and differentiation, thus benefiting for the tissue repair and regenerative medicine for the last two decades. To improve the mechanical characteristics, physicochemical and biological properties, CS-based hydrogels are required to combine with other natural or synthetic polymers, which can create a series of environmentally responsive and injectable hydrogels for biomedical engineering applications. In addition, incorporation of bioactive molecules with CS-based hydrogels can induce the angiogenesis and vascularization, accelerate bone repair and promote periodontal tissue engineering. We believe that further research on chitosan and the search for new variations in its use with other polymers will reveal greater prospects in biomedical applications.

Although CS-based hydrogels are possible alternative for current clinical practices of bone defect repair with vast array of beneficial properties, there is some clinical limitation for bone and dental regeneration. One major limitation is the standardization of molecular weight distribution, raw material resources and commercial production. High molecular weight may cause potential inflammation as a bone graft, and cell wall-derived CS may be more reliable than that from seafood due to the varieties and reproducibility of fungal and marine sources all over the world. In addition, CS-based hydrogels as bone scaffolds are their infancy in experimental research with limited studies and insufficient clinical utility. Therefore, further research should be focused on the establishment of uniform standards and invention of advanced technology to expand potential clinical applications. Additionally, improvement of higher loading capacity and controlled release of bioactive molecules into CS-based hydrogels will be a prime research objective upon implantation for clinical applications. Going forward clinical research is the combination of CS-based hydrogels with different biofabrication techniques like electrospinning, microspheres and 3D-printing for building a multifaceted and multilayered bone graft in bone regeneration and fracture management.

Thus, we believed that along with clear elucidation of the molecular and signaling mechanisms on bone and dental regeneration, CS-based hydrogels will spark broader interests in the scientific community to create more tailormade tissue-engineering scaffolds with optimum characteristics and advanced properties like natural bones to combat large bone defects in clinical therapeutics.

## Author Contributions

RC, XW, and XY initiated the project. GT, ZT, WZ, CS, YL, and HH searched the data base. GT, XW, and XY wrote, revised, and finalized the manuscript. All authors contributed to the article and approved the submitted version.

## Conflict of Interest

The authors declare that the research was conducted in the absence of any commercial or financial relationships that could be construed as a potential conflict of interest.

## Abbreviations

ACP: amorphous calcium phosphate
ALN: alendronate
ASP: aspirin
BCP: biphasic calcium phosphate
β-GP: β-sodium glycerophosphate
bFGF: basic fibroblast growth factor
BMPs: morphogenetic proteins
BMP-2: bone morphogenetic protein 2
BMP-7: bone morphogenetic protein-7
BTE: bone tissue engineering
CMCh: carboxymethyl chitosan
CS: chitosan
DPSCs: dental pulp stem cells
ECM: extracellular matrix
EPO: erythropoietin
GP: β-glycerophosphate
MCS: maleilated chitosan
MSCs: mesenchymal stem cells
NIPAAm: N-isopropylacrylamide
NPs: nanoparticles
nBG: bioactive glass NP
ORN: ornidazole
PEGDA: poly(ethylene glycol) diacrylate
PVA: poly(vinyl alcohol)
VEGF: vascular endothelial growth factor.
